# Moderate-Intensity Physical Exercise Affects the Exercise Performance and Gut Microbiota of Mice

**DOI:** 10.3389/fcimb.2021.712381

**Published:** 2021-09-24

**Authors:** Wenqian Yang, Yuqian Liu, Guang Yang, Binglin Meng, Zhicheng Yi, Guan Yang, Mingjian Chen, Pengcheng Hou, Haitao Wang, Xiaoyang Xu

**Affiliations:** ^1^ School of Physical Education and Sports Science, South China Normal University, Guangzhou, China; ^2^ School of Physical Education, Guangdong Baiyun University, Guangzhou, China; ^3^ School of Sport & Exercise Science, Lingnan Normal University, Zhanjiang, China; ^4^ Department of Basic Education, Guangzhou Vocational and Technical University of Science and Technology, Guangzhou, China; ^5^ Training & Scientific Research & Medical Management Center, Hebei Institute of Sports Science, Shijiazhuang, China; ^6^ School of Physical Education, South China University of Technology, Guangzhou, China

**Keywords:** exercise intervention, dominant genera, core bacteria, alpha diversity, 16S rRNA

## Abstract

The gut microbiota is closely associated with the health of the host and is affected by many factors, including exercise. In this study, we compared the gut microbial changes and exercise performance over a 14-week period in mice that performed exercise (NE; n = 15) and mice that did not perform exercise (NC; n = 15). Mice were subjected to stool collection and exercise tests one week prior to adaptive training and after 2, 6, 10, and 14 weeks of exercise. Bacteria associated with the stool samples were assessed *via* Illumina-based 16S rRNA gene sequencing. While there was no significant difference in body weight between the groups (p > 0.05), the NE group had a significantly higher exercise performance from weeks 2–14 (p < 0.01) and lower fat coefficient (p < 0.01) compared with the NC group. The Shannon index of the gut microbiota in the NC group was higher than that in the NE group at weeks 6 and 10, and the Chao1 index was higher than that in the NE group at week 14. Exercise performance positively correlated with the relative abundance of Phascolarctobacterium. Grouped time series data analysis demonstrated that Bifidobacteria, Coprococcus, and one unnamed genus in the Clostridiales order were significantly increased in the NE group, which correlated with increased glucose, flavonoid, arginine, and proline metabolism. In conclusion, moderate-intensity treadmill exercise significantly increased the exercise performance of mice and changed the core bacteria and bacterial metabolic activity. These results provide a reference for studying the effects of exercise intervention and exercise performance on the gut microbiota of mice.

## Introduction

The gut microbiota plays an essential role in human health (Diaz Heijtz, 2016) and is involved in several physiological functions of the host, including detoxification, digestion, metabolism, production of nutrients, regulation of the immune system, protection against pathogens, and development of intestinal epithelial cells (Eckburg et al., 2005; Ley et al., 2006; Hill and Artis, 2010). Additionally, disruptions to the gut microbiota have been linked to a number of chronic intestinal conditions, such as inflammatory bowel disease (Frank et al., 2007; Morgan et al., 2012), as well as a variety of systemic diseases, such as obesity (Turnbaugh et al., 2006; Zhang et al., 2009; Clarke et al., 2012), type 2 diabetes (Qin et al., 2012), and cancer (Zitvogel et al., 2015). Thus, the gut microbiota plays a fundamental role in the well-being of the host.

There is growing evidence that the gut microbiota can be modulated by various factors such as infection, antibiotics, diet, and exercise (Grenham et al., 2011). Exercise—the planned, structured, and repetitive physical activity that is performed with the purpose of improving or maintaining physical fitness (Caspersen et al., 1985)—has been shown to influence the gut microbiota and microbiome in rats (Matsumoto et al., 2008). Both exercise and a high-fiber diet have been reported to increase the diversity of the gut microbial community (Clarke et al., 2014). Additionally, recent studies have suggested that the number of beneficial microbe species is enhanced by formal exercise (Ostrowski et al., 1987). It has also been shown that the gut microbiota can respond to both homeostatic and exercise-induced physiological variations, and that exercise changes the composition of the gut microbiota by playing a positive role in energy homeostasis (Ostrowski et al., 1987). Notably, exercise-induced changes in the gut microbiome have been correlated with corresponding changes in host physiology, including immunity and metabolism (Queipo-Ortuño et al., 2013).

Exercise has been shown to improve both the physical and mental health of persons of any age (Bell et al., 2019), and is also often included in treatment plans for many chronic diseases (Pedersen and Saltin, 2015). Moreover, previous studies have indicated that exercise-induced metabolic effects have the capability to positively reshape the gut microbiota in various animal models as well as humans (Codella et al., 2018). However, changes to the structure of the gut microbiota vary depending on the exercise intensity, mode, and time (Ribeiro et al., 2019; Mohr et al., 2020).

Thus, the purpose of our study was to determine if a 14-week, moderate-intensity exercise program could favorably modify the intestinal microbiota and influence the exercise performance of mice. The energy consumption of moderate-intensity exercise is 3–6 times higher than that of basic metabolism, and the intensity range is close to 40%–59% of the heart rate reserve or oxygen uptake reserve, 64%–76% of the maximum heart rate, and 46%–63% of the maximum oxygen uptake (ACSM, 2010). In this study, the training plan was made according to the experimental purpose and the relevant literature (Schefer and Talan, 1996; Kwon et al., 2014; Ma et al., 2019). The purpose of this study was to investigate the relationship between exercise performance and gut microbiota. Hence, the training needs to be sufficient to improve exercise performance. Low-intensity exercise has no substantial effect on improving exercise performance, while high-intensity exercise training may lead to intestinal function impairment, gut microbiota and metabolism disorders and other adverse effects (Feng and Cao, 2016).

Most of the previous studies have focused on observing the changes in microbiota and metabolism after training (Allen et al., 2018; Ribeiro et al., 2019), while this study focused on the changes in microbiota and metabolism during the process of improving exercise performance through training. The results of this study can provide reference for improving exercise performance, and the relationship between the changes in microbiota and metabolism and exercise performance found in the training process can provide ideas for improving exercise performance with gut microbiota as the entry point in the future.

## Materials and Methods

### Experimental Approach

We compared the gut microbiota and exercise performance of mice subjected to no exercise or moderate-intensity exercise over a 14-week period. We collected stool and subjected both groups of mice to exercise performance tests at 5 different time points throughout the experiment, in addition to determining daily food intake and taking body weight measurements weekly. Bacteria associated with the stool samples were assessed *via* 16S rRNA sequencing.

### Subjects

Thirty specific pathogen-free C57BL/6 male mice (5 weeks old; 18 ± 2 g) were purchased from the Guangdong Medical Experimental Animal Center (Guangdong, China). Upon arrival at our facility, the animals were housed in groups of 3–4 mice per cage in a room with a 12-hour light/dark cycle, an average temperature of 20–25°C, and relative humidity of 50–70%. The mice had access to water and regular chow (Guangdong Medical Experimental Animal Center, Guangdong, China) ad libitum. Components of the regular chow included corn, soybean meal, flour, wheat sub-meal, Peruvian fish meal, calcium hydrogen phosphate, stone powder, sodium chloride, vegetable oil, vitamins, amino acids, and minerals. All animal experiments were approved by the Scientific Research Ethics Sub-committee, School of Physical Education, South China Normal University (Guangzhou, China) (approval number: SCNU-SPT-2019-002).

### Procedures

#### Experimental Grouping and Exercise Intervention Program

After acclimating to the vivarium for two weeks, the mice were randomly divided into a control group (NC group) and exercise group (NE group), with 15 mice in each group. The food intake was calculated daily. The added food was weighed with an electronic balance (Zhongshan Jiawei Trading Co., Ltd., Guangdong, China) at 6 PM daily and 5 g of food was added per mouse per day. The next day, the remaining food was weighed and subtracted from the added amount, to determine the daily food intake. The mice were observed daily for signs of illness including a ruffled coat and injuries from fighting. Aggressive mice were separated into different cages, and open cuts from fighting were immediately cleaned with iodine (Ali Health Drugstore Pharmaceutical Chain Co., Ltd., Guangdong, China) every day for a week. Injured mice were removed from the study. The mice were weighed at the same time every week.

For the exercise intervention program, a rodent treadmill (Guangzhou Feidi Biological Technology Co., Ltd., Guangdong, China) was used. The mice in the NE group were subjected to adaptive treadmill exercise (3 days a week, 10–20 minutes a day, slope 0, speed 10–13 m•min^−1^), followed by formal exercise training for 14 weeks. Formal exercise training was conducted with moderate-intensity treadmill exercise for 60 minutes a day, 5 days a week, with a slope of 0. The speed in weeks 1–2 was 14 m•min^−1^, the speed in weeks 3–6 was 15 m•min^−1^, the speed in weeks 7–10 was 17 m•min^−1^, and the speed in weeks 11–14 was 19 m•min^−1^. To eliminate the impact of other environmental factors on the results, the mice in the NC group were exposed to the same experimental environment during exercise training, but were not subjected to treadmill exercise.

#### Fecal Sample Collection Schedule and Method

The initial fecal samples were collected from the NC and NE groups before adaptive training. Fecal samples were then collected from the NC and NE groups at 2, 6, 10, and 14 weeks after the initiation of formal exercise. Thus, there were 5 sampling points for both groups. NC samples were recorded as NC0, NC2, NC6, NC10, and NC14. NE samples were recorded as NE0, NE2, NE6, NE10, and NE14.

Mice were placed in a clean cage (1 mouse per cage) with aseptic filter paper (Yancheng Haikuo Experimental Equipment Co., Ltd., Jiangsu, China) on the bottom. A cotton swab was used to rub the abdomen and anus of the mice to stimulate defecation. Approximately five fecal pellets were collected from each mouse. Fecal pellets were collected with sterile forceps (Shanghai Rebus Biotechnology Co., Ltd., Shanghai, China), placed in a sterile cryovial (Shanghai Rebus Biotechnology Co., Ltd.) (one pellet per tube), and stored on dry ice. All fecal pellets were stored at −80°C until future use.

#### Exercise Performance Test

To determine the exercise performance of mice, the longest running distance of exhaustive exercise was measured. The mice in both the NC and NE groups underwent a total of five exercise performance tests over the course of the experiment. Exercise performance tests were performed at the same time for both the NC and NE groups. Since mice in the NC group were not exposed to exercise training, these mice underwent adaptive training (10–20 minutes a day, slope 0, speed 10–13 m•min^−1^) for three consecutive days prior to each exercise performance test. The first exercise performance test for both the NC and NE groups occurred after the end of adaptation period. The other tests occurred at weeks 2, 6, 10, and 14 of the formal exercise intervention.

The mice were fasted for 2 hours before the exercise performance test. The mice were placed on the small animal running platform set at a slope of 0. The treadmill speed for the exercise performance test started at 10 m•min^−1^, then was increased to 12 m•min^−1^, and then was increased in 1 m•min^−1^ increments per minute until the speed reached 21 m•min^−1^. Once a constant speed of 21 m•min^−1^ was reached, the speed was increased 1 m•min^−1^ every 20 minutes until exhaustion or 120 minutes. The exhaustion time and speed were recorded. The total running distance of each mouse was calculated according to time and speed. Mouse exhaustion was judged according to the following standards: could not maintain the predetermined running speed, the running posture was deformed, lagged behind the track for an extended period of time, mouse was short of breath, looked tired, and showed an abdominal lying position after being removed from the treadmill. Mice that were not exhausted were able to maintain a good exercise posture, typically did not stay at the back 1/3 of the platform, and accelerated their running with a little pressure. The maximum running distance of the mice was recorded as the exercise performance of the mice.

#### Detection of Gut Microbiota

DNA Extraction: Total bacterial genomic DNA was extracted from fecal stool pellets using the PowerMax (stool/soil) DNA isolation kit (MoBio Laboratories, Carlsbad, CA, USA). The quantity and quality of extracted DNA were measured using a NanoDrop ND-1000 spectrophotometer (Thermo Fisher Scientific, Waltham, MA, USA) and agarose gel electrophoresis, respectively.

16S Amplicon Pyrosequencing: PCR amplification of the bacterial 16S rRNA genes V4 region was performed using the forward primer 515F (5’-GTGCCAGCMGCCGCGGTAA-3’) and the reverse primer 806R (5’-GGACTACHVGGGTWTCTAAT-3’). Sample-specific, paired-end, 6-bp barcodes were incorporated into the TrueSeq adaptors for multiplex sequencing. PCR amplicons were purified with Agencourt AMPure XP Beads (Beckman Coulter, Indianapolis, IN, USA) and quantified using the PicoGreen dsDNA Assay Kit (Invitrogen, Carlsbad, CA, USA). After the individual quantification step, amplicons were pooled in equal amounts, and pair-end 2 × 150 bp sequencing was performed using the Illlumina NovaSeq6000 platform at Hangzhou Guhe Information Technology Co., Ltd. (Hangzhou, China).

Sequence Analysis: The Quantitative Insights Into Microbial Ecology (QIIME, v1.9.0) **(** Caporaso et al., 2010 **) **pipeline was employed to process the sequencing data.

Bioinformatics and Statistical Analysis: Sequence data analyses were mainly performed using the QIIME and R packages (v3.2.0). Operational taxonomic units (OTU)-level alpha diversity indices, such as Chao1 richness estimator, ACE metric, Shannon diversity index, and Simpson index, were calculated using the OTU table in QIIME. OTU-level ranked abundance curves were generated to compare the richness and evenness of OTUs among samples. Beta diversity analysis was performed to investigate the structural variation of the microbial communities. Principal coordinate analysis (PCoA) is a visualization method used to study the similarities or differences in data. PCoA can identify the most important coordinates in the distance matrix. The result is a rotation of the data matrix, which does not change the position relationship between the sample points (Jolliffe and Cadima, 2016). Through PCoA, differences between individuals or groups can be observed (Jolliffe and Cadima, 2016). Differences in the UniFrac distances for pairwise comparisons among groups were determined using Student’s *t* test and the Monte Carlo permutation test with 1,000 permutations and were visualized *via* box-and-whiskers plots. Taxa abundances at the phylum, class, order, family, genus, and species levels were statistically compared among samples or groups *via* Kruskal test using the R stats package. Linear discriminant analysis effect size (LEfSe) was performed to detect differentially abundant taxa across groups using the default parameters of the linear discriminate analysis (LDA) score (default 2.0). The time intervals of the mutually differentially abundant genera from groups identified by METAgenomic LONgitudinal Differential Abundance method (MetaLonDA). Microbial functions were predicted by PICRUSt (Phylogenetic investigation of communities by reconstruction of unobserved states) based on high-quality sequences. In addition, a Pearson correlation analysis was conducted between exercise capacity and the relative abundance of bacteria. Correlation clustering analysis by hierarchical all-against-all association (HALLA) was used to discover significant relationships between data features at high power. The output file was further analyzed using Statistical Analysis of Metagenomic Profiles (STAMP) software package v2.1.3.

#### Tissue Collection

The mice were euthanized 48 hours after the last exercise performance test. The mice were fasted 12 hours prior to euthanasia. The mice were weighed immediately before euthanizing, and the body length (distance from tip of nose to anus) of each mouse was measured afterwards. The mice were perfused with PBS, and then the epididymal fat, perirenal fat, groin fat, tibialis anterior muscle, gastrocnemius and quadriceps femoris were harvested, weighed, and snap-frozen in liquid nitrogen. The samples were stored at −80°C until future use.

### Statistical Analysis

The data were statistically analyzed using SPSS 20.0 software (Chicago, IL, USA) and R. All data were expressed as mean ± standard deviation. Independent-sample *t* test, repeated-measures ANOVA, rank-sum test, LEfSe, and MetaLonDA were used to compare groups, and correlation analysis was also conducted. p < 0.05 was considered statistically significant.

## Results

### Effects of Exercise on Food Intake and Body Weight

**Figure 1:**
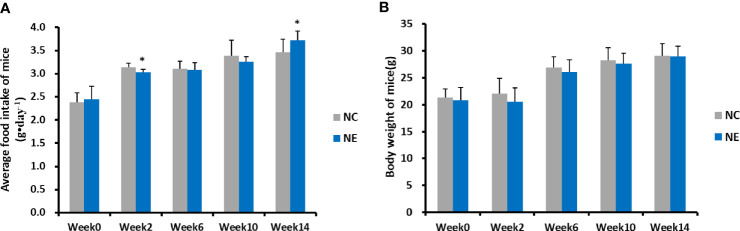
Average food intake of mice (g•day^−1^) and body weight of mice (g). Repeated-measures ANOVA was used to compare the groups. **(A)** average food intake of mice (g•day^−1^). **(B)** body weight of mice (g). NC = control group; NE = exercise group. Compared with the NC group, *p < 0.01.

The food intake and body weight of mice in both groups was measured over 14 weeks and analyzed using repeated-measures ANOVA (**Figure 1**). For the food intake (**Figure 1A**), result of Mauchly’s test of sphericity showed p < 0.05, the results of multivariate tests (Roy’s Largest Root) showed that the main effect of time was significant (F = 682.925, p = 0.000). There were significant differences in food intake between different time points, and food intake increased with the extension of intervention time. The interaction effect of time and group was significant (F = 86.435, p = 0.000), indicating that the food intake trend was different among different groups of mice. Simple-effect analysis showed that the food intake of mice in NC group and NE group at 2 weeks, 6 weeks, 10 weeks, and 14 weeks was significantly higher than that before intervention (p < 0.01), showing a gradually increasing trend. At week 2, food intake in NC group was significantly higher than that in NE group (p < 0.01); at week 14, food intake in NC group was significantly lower than that in NE group (p < 0.01). There was no significant difference in food intake between the two groups at other time points. As for body weight (**Figure 1B**), the result of Mauchly’s test of sphericity showed p < 0.05. The results of multivariate tests (Roy’s Largest Root) showed that the main effect of time was significant (F = 117.862, p = 0.000). There was a significant difference in body weight between different time points; namely, the body weight of mice increased with the intervention time. The interaction effect of time and group was not significant (F = 1.444, p = 0.249), showing that the effect of time was similar among the groups. The tests of between-subjects effects showed no significant difference in body weight between the two groups during the entire experimental time frame (F = 0.975, p = 0.332).

### Body Length, Lee’s Index, Muscle Content(%) and Fat Content(%)

**Figure 2:**
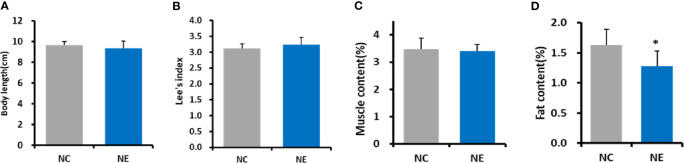
Body length, Lee’s index, muscle content (%), and fat content (%). Independent-samples *t* test were used to compare the groups. **(A)** body length. **(B)** Lee’s index=body weight (g)×10003body length (cm) ; **(C)** Muscle content (%) = [tibialis anterior muscle (g) + gastrocnemius (g) + quadriceps femoris (g)]/body weight (g) * 100; **(D)**, Fat content (%) = [perirenal fat (g) + inguinal fat (g) + epididymal fat (g)]/body weight (g) * 100. NC = control group; NE = exercise group. Compared with the NC group, *p < 0.01.

We estimated the obesity in the mice *via* Lee’s index. Lee’s index=body weight (g)×10003body length (cm) is a commonly used method to judge obesity and body weight in small experimental animals, such as mice **(** Bernardis and Patterson, 1968; Rogers and Webb, 1980 **)**. Independent-sample *t* test was used to analyze the differences between the groups. There were no significant differences in body length, Lee’s index or muscle content between NE group and NC group (**Figures 2A–C**). Furthermore, we also measured the fat content in the mice of both groups. We found that the fat content in NE group was significantly lower than that in NC group (p < 0.01) (**Figure 2D**).

### Effect of Moderate-Intensity Exercise on the Exercise Performance

**Figure 3:**
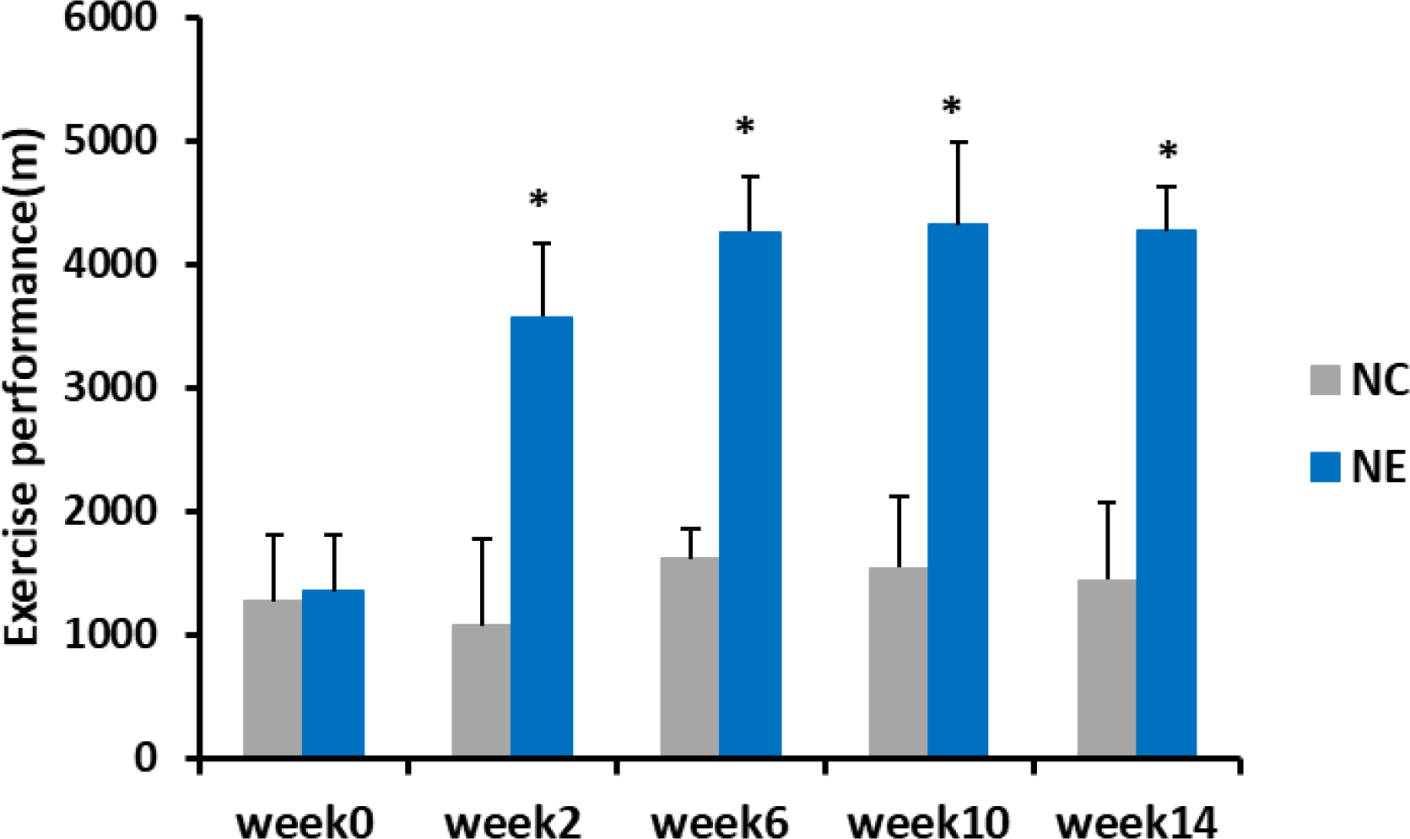
Exercise performance of mice (m). Repeated-measures ANOVA was used to compare the groups. NC = control group; NE = exercise group. Compared with the NC group, *p < 0.01.

To determine how moderate-intensity exercise over an extended period affects the exercise performance of mice, we recorded the maximum running distance of control and exercise-subjected mice five times over 14 weeks. Repeated-measures ANOVA was used to analyze changes in exercise performance in mice. The result of Mauchly’s test of sphericity showed p > 0.05. The tests of within-subjects effects showed that the main effect of time was significant (F = 53.412, p = 0.000). Over time, the exercise performance changed significantly. The interaction between time and group was significant (F = 37.945, p = 0.000), indicating that the exercise performance trend of different groups of mice was different. Simple-effects analysis showed no significant differences in the exercise performance of NC group between the time points. The exercise performance before intervention of NE group was significantly lower than that at other time points (p < 0.01). The exercise performance of NE group increased significantly at weeks 2 and 4, but not significantly from week 6. Before intervention, there was no significant difference in the exercise performance between NC and NE groups. However, from week 2, the exercise performance of NE group was significantly higher than that of NC group (p < 0.01) (**Figure 3**).

### Effect of Exercise on the Composition of Gut Microbiota and Diversity Analysis

#### Original Sequence Data and Quality Control

The data was obtained in fastq format, and then the samples were paired and spliced into a single sequence. The sample corresponding to a sequence was determined by barcode, and the sequences were filtered by QIIME software (Quantitative Insights Into Microbial Ecology, v1.8.0, http://qiime.org/). Chimeras were removed to obtain the effective sequences.

As shown in **Supplementary File Table 1**, a total of 20,434,356 tags were attained from the 150 sequencing samples in this study, with an average of 136,229 tags per sample (range = 66,738–139,999, SD = 13,478). We then obtained 19,742,005 clean_tags, with an average of 131,613 per sample (range = 57,504–138,463, SD = 14,673). Among these, 618,220 operational taxonomic units (OTUs) were obtained, with an average of 4121 per sample (range = 1,207–6,654, SD = 1,192). The 16S sequencing depth of samples in this experiment reached approximately 100,000 reads. Therefore, the data volume of tags and clean tags met the sequencing requirements. The Good’s coverage for the sequencing reads was 98.91%.

#### Dilution Curve and Shannon Curve

**Figure 4:**
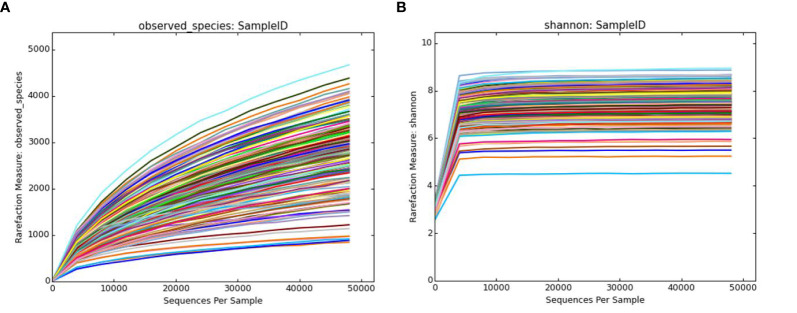
Dilution curve and Shannon-Wiener curve. **(A)** The observed_species dilution curve. The observed_species index represents the actual number of OTUs observed. The x-axis represents the number of sequences randomly selected for each sample, and the y-axis represents the number of OTUs at the corresponding depth. **(B)** The Shannon-Wiener curve. The x-axis represents the number of randomly selected sequences, and the y-axis represents the Shannon index that reflects the species diversity. Each color in the figure represents a single sample. OTU, operational taxonomic unit.

As shown in **Figure 4A**, new germline types may be identified as the amount of sequencing increases. As shown in **Figure 4B**, the current sequencing depth covered the majority of the bacterial diversity in the sample. Thus, the sequencing depth in this study was able to fully display information regarding bacterial abundance within the samples.

#### Analysis of the Composition of the Gut Microbiota at Different Taxonomic Levels

**Figure 5:**
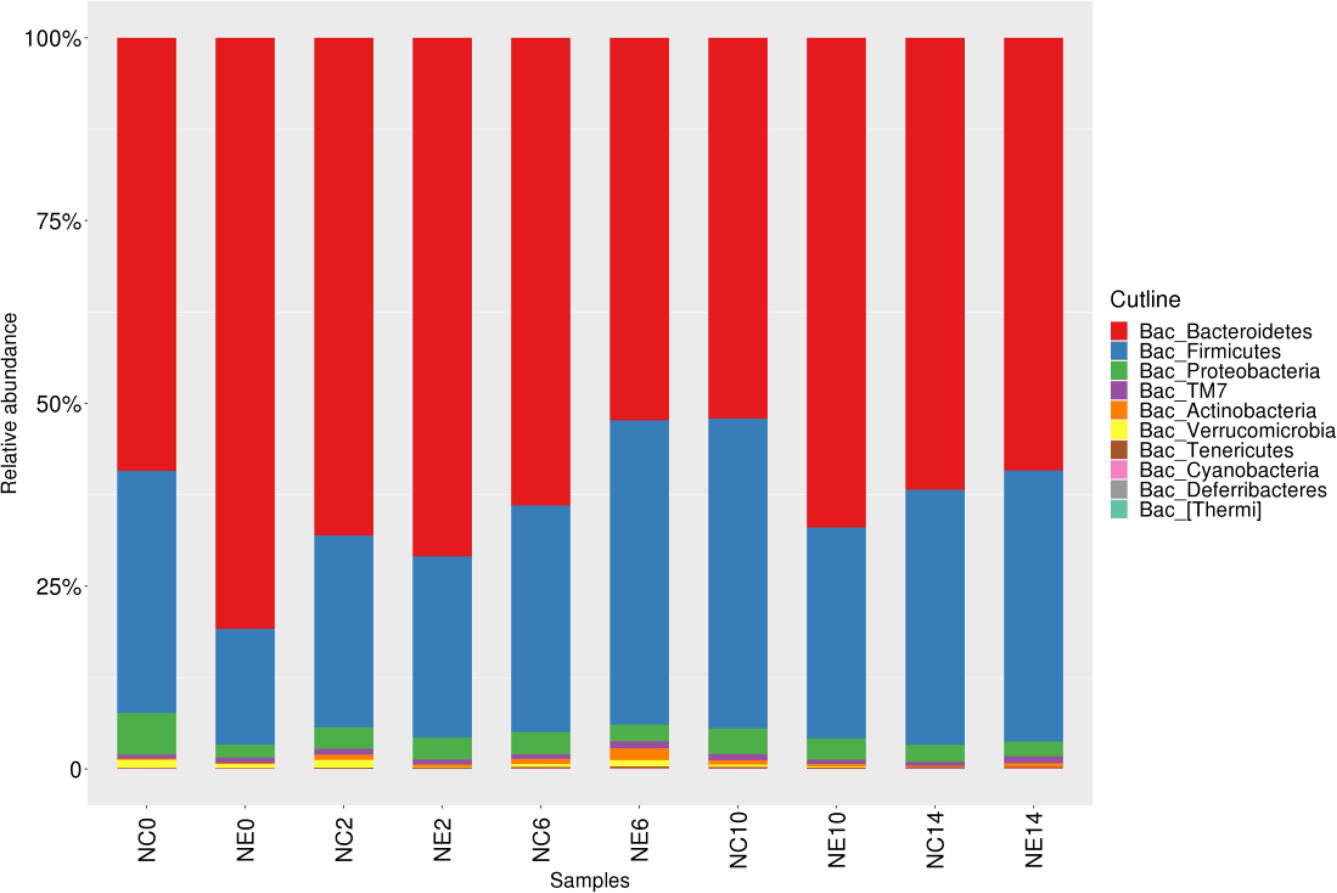
Histogram of the gut microbiota at the phylum level in the control (NC) and exercise (NE) groups at each time point. The ordinate represents the relative abundance of phyla. Different colors represent different phyla.

Through clustering and OTU annotations, we obtained the relative abundance of microbial communities in each group of samples at different classification levels. A total of 11 phyla were identified, of which the three phyla with the highest relative abundance were Bacteroidetes (63.14%), Firmicutes (31.80%), and Proteobacteria (3.03%). The relative abundance of these three phyla accounted for 97.97% of the total sequence. However, TM7, Actinobacteria, Verrucomicrobia, Tenericutes, undefined phylum, Cyanobacteria, Deferribacteres, and Thermi decreased (**Figure 5**). In both the NC and NE groups, the dominant phyla were Bacteroidetes, Firmicutes, and Proteobacteria.

**Figure 6:**
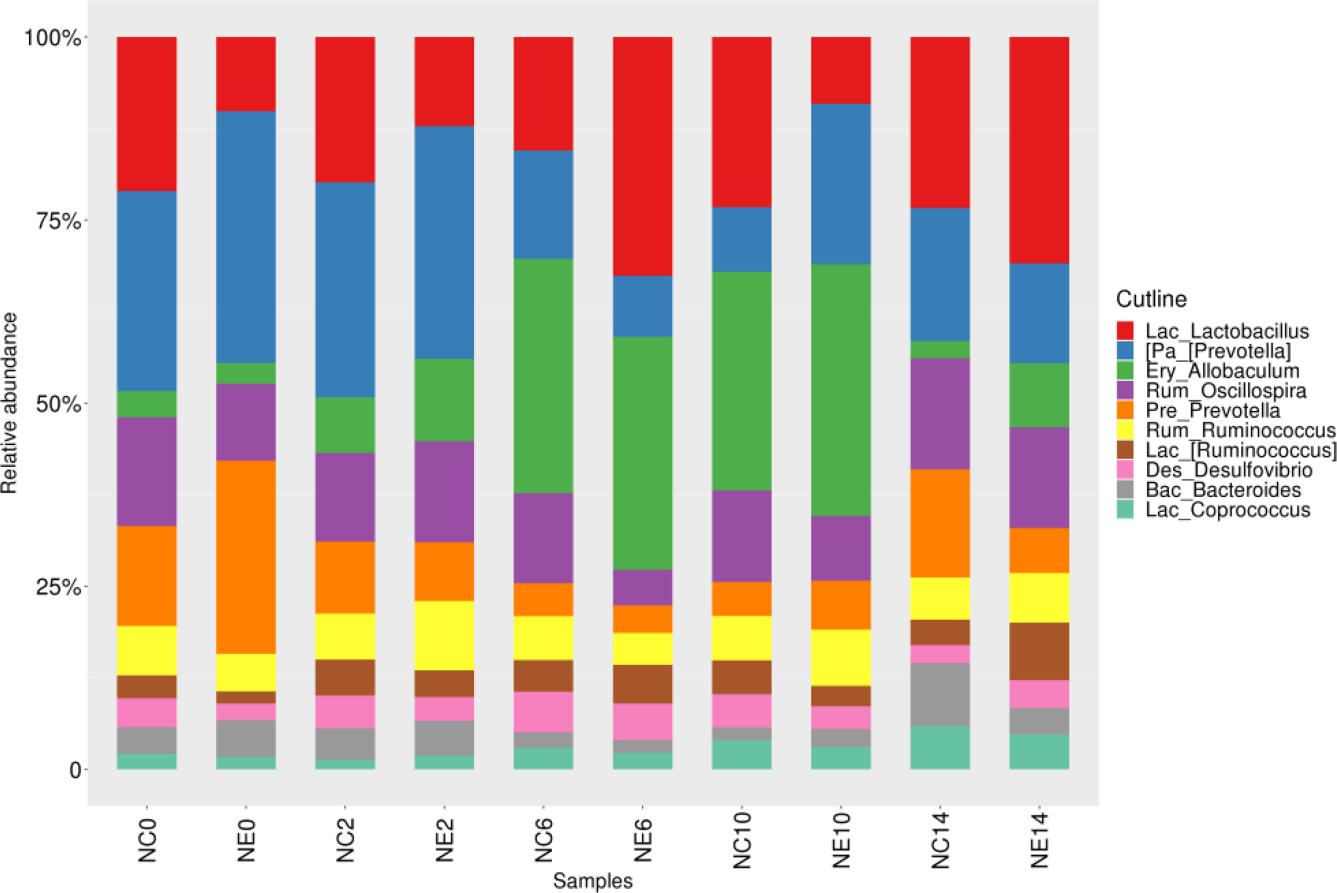
Histogram of the gut microbiota at the genus level in the control (NC) and exercise (NE) groups at each time point. The ordinate represents the relative abundance of genera. Different colors represent different genera.

The common dominant genera of each group were *Lactobacillus* (3.5%), *Allobaculum* (3.1%), *Oscillospira* (2.1%), *Ruminococcus* (1.8%), *Coprococcus* (0.5%; Firmicutes phylum), *Prevotella* (5%), *Bacteroides* (0.6%; Bacteroidetes phylum), and *Desulfovibrio* (0.7%; Proteobacteria phylum) (**Figure 6**). The dominant genera in the NC group included *Lactobacillus* (3.2%), *Oscillospira* (2.4%), *Allobaculum* (2.4%), *Ruminococcus* (1.8%; Firmicutes phylum), *Prevotella* (5.2%), *Bacteroides* (0.7%; Bacteroidetes phylum), *Desulfovibrio* (0.8%; Proteobacteria phylum), and *Akkermansia* (0.6%; Verrucomicrobia (**Supplementary File Figure 1**). The dominant genera in the NE group were *Lactobacillus* (3.9%), *Allobaculum* (3.7%), *Ruminococcus* (1.8%), *Oscillospir* (1.7%), *Coprococcus* (0.5%; Firmicutes phylum), *Prevotella* (4.9%), *Bacteroides* (0.6%; Bacteroidetes phylum), and *Desulfovibrio* (0.6%; Proteobacteria phylum) (**Supplementary File Figure 2**).

Wilcoxon rank-sum test (BH-corrected) was used to compare the relative abundance of bacteria between the groups. The results showed that four phyla differed between the groups before intervention. The relative abundance of Bacteroidetes in NC group was significantly lower than that in NE group (p < 0.01), while the relative abundances of Firmicutes, Proteobacteria, and TM7 in NC group was significantly higher than that in NE group (p < 0.01, p < 0.01, and p < 0.05, respectively). At week 2, the relative abundance of Actinobacteria in NC group was significantly higher than that in NE group (p < 0.05), and at week 14, that in NC group was significantly lower than that in NE group (p < 0.05). Before intervention, there were five bacterial genera with differential relative abundance. The relative abundances of *Lactobacillus*, *Dehalobacterium*, *Lac_[Ruminococcus]*, *Rum_Ruminococcus*, and *Desulfovibrio* in NC group were significantly higher than those in NE group (p < 0.01, p < 0.05, p < 0.01, p < 0.05, p < 0.05). At week 2, the relative abundance of *Dorea* in NC group was significantly lower than that in NE group (p < 0.05); At week 6, the relative abundances of *Dehalobacterium* and *Oscillospira* in NC group were significantly higher than those in NE group (p < 0.05). At week 10, the relative abundance of *Lactobacillus* in NC group was significantly lower than that in NE group (p < 0.05), and the relative abundances of *Oscillospira* and *Desulfovibrio* in NC group was significantly higher than that in NE group (p < 0.05). At week 14, the relative abundances of *Bacteroides*, *Parabacteroides*, and *Odoribacter* in NC group were significantly higher than those in NE group (p < 0.05).

#### Box Diagram of Alpha Diversity Index Difference

**Figure 7:**
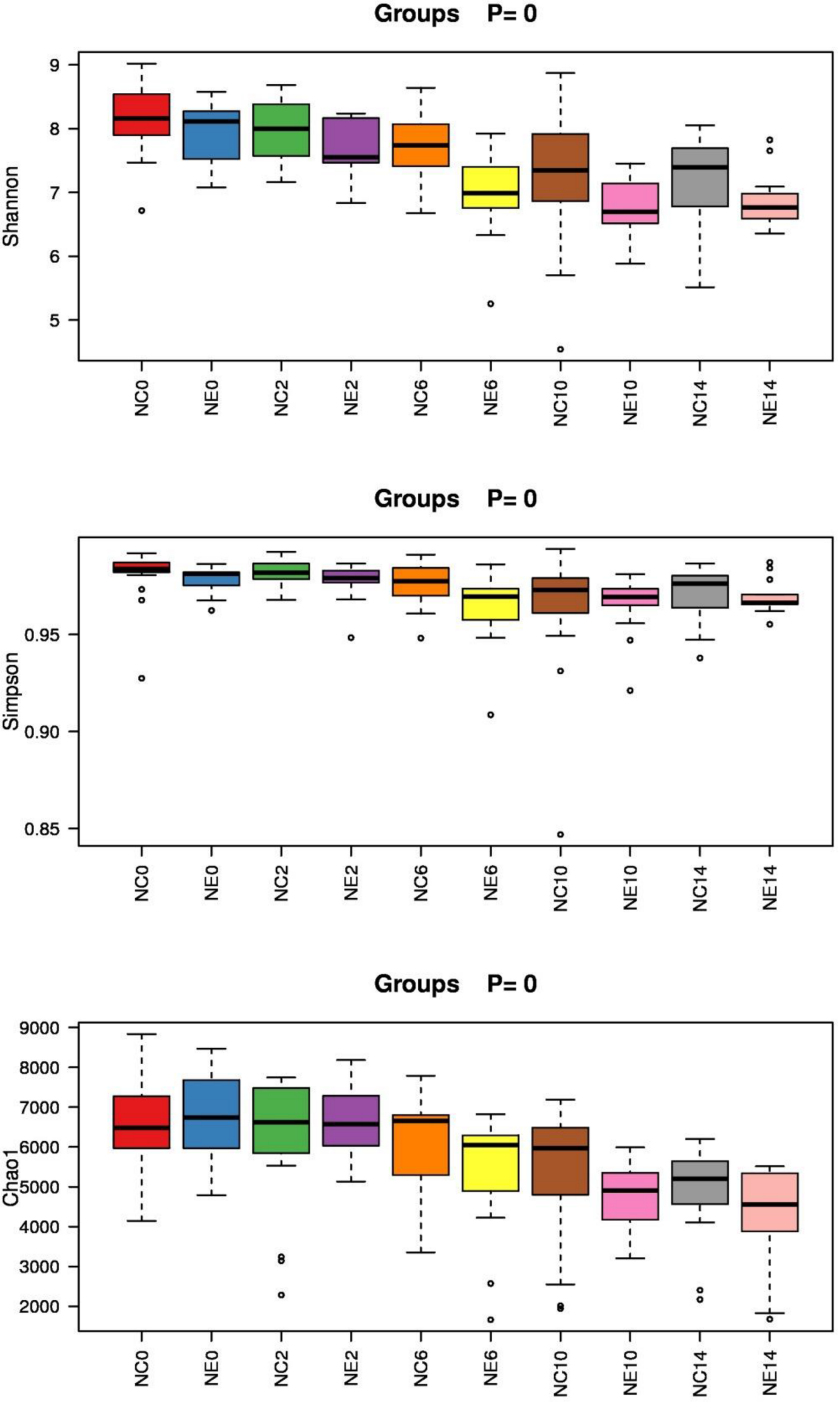
Box diagram of the alpha diversity index of each group of samples. Shannon index, Simpson index, and Chao1 index. The x-axis is the group name, and the y-axis is the value of the alpha diversity index in different groups. The box chart shows five statistics (minimum, first quartile, median, third quartile, and maximum). The abnormal value is marked with an “o”, and the top is marked with the *p*-value (Kruskal-Wallis H-rank sum test). NC = control group; NE = exercise group.

After determining that the sequencing depth was sufficient, we first evaluated the alpha diversity of the samples. Each index of alpha diversity was analyzed by Kruskal-wallis H-rank sum test. (). The alpha diversity index with significant differences under different conditions was screened by rank sum test. The Shannon index, Simpson index, and Chao1 box chart of each group are shown in **Figure 7**.

In addition, repeated-measures ANOVA was used to analyze changes in Shannon index in mice. The result of Mauchly’s test of sphericity showed p > 0.05. The tests of within-subjects effects showed that the main effect of time was significant (F = 17.188, p = 0.000). Over time, the Shannon index changed significantly. There was no significant interaction between time and group (F = 1.075, p = 0.309), suggesting that the Shannon index of different groups showed the same trend over time. The results of tests of between-subjects effects showed significant differences in Shannon index among different groups (F = 10.042, p = 0.004). Before intervention and at weeks 2 and 14, there were no significant differences between the groups, but at weeks 6 and 10, Shannon index of NC group was significantly higher than that of NE group (t = 2.319, p = 0.028; t = 2.203, p = 0.036).

Repeated-measures ANOVA was used to analyze changes in Simpson index in mice. The result of Mauchly’s test of sphericity showed p < 0.05. The results of multivariate tests (Roy’s Largest Root) showed that the main effect of time was significant (F = 4.460, p = 0.007). There were significant differences in Simpson index between different time points. There was no significant interaction between time and group (F = 0.702, p = 0.598), indicating that the Simpson index of different groups showed the same trend over time. The results of tests of between-subjects effects showed that the groups differed in the Simpson index (F = 5.090, p = 0.032); however, there was no significance difference among the time points (p > 0.05).

Repeated-measures ANOVA was used to analyze changes in Chao1 index in mice. The result of Mauchly’s test of sphericity showed p < 0.05. The results of multivariate tests (Roy’s Largest Root) showed that the main effect of time was significant (F = 4.000, p = 0.000). There were significant differences in Chao1 index between different time points. There was no significant interaction between time and group (F = 4.000, p = 0.391), indicating that the Simpson index of different groups showed the same trend over time. The result of tests of between-subjects effects showed differences in the Chao1 index between the groups (F = 6.214, p = 0.019). Before intervention, and at weeks 2, 6, and 10, there were no significant differences between the groups, but at week 14, Chao1 index of NC group was significantly higher than that of NE group (t = 2.319, p = 2.228; t = 2.203, p = 0.034).

#### Beta Diversity Analysis

**Figure 8:**
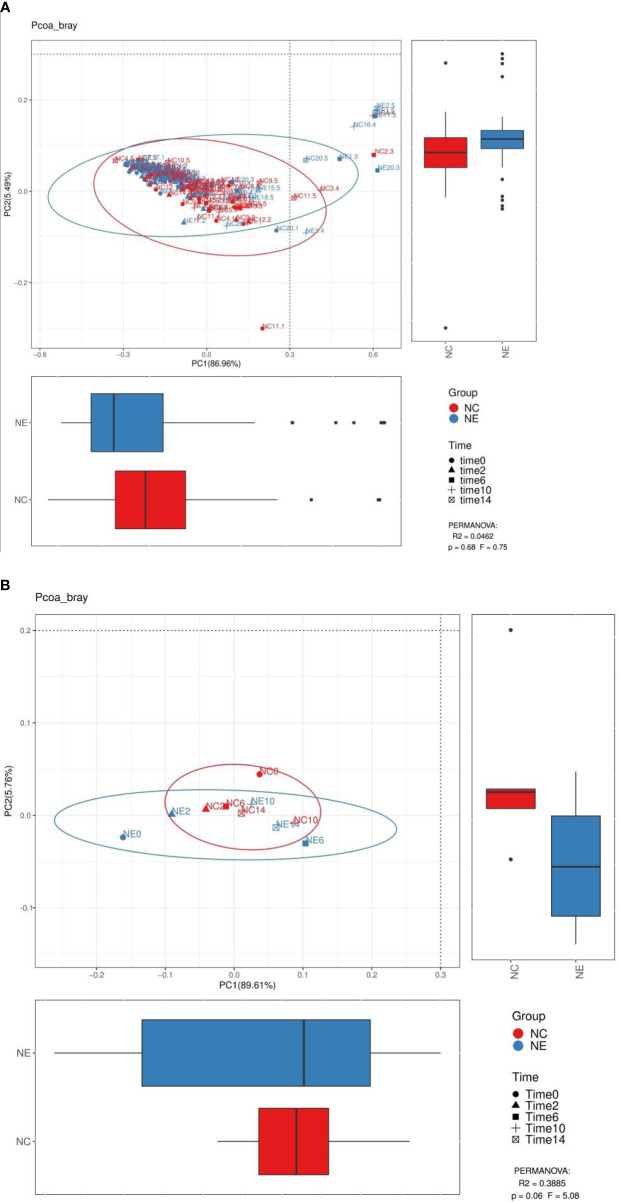
Analysis diagram of principal coordinate analysis (PCoA) at the phylum level. We performed PCoA analysis based on Bray-Curtis distance. Each point in **(A)** represents a sample, and each point in **(B)** represents the average of 15 samples at a point in time. The points of the same color come from the same group, and the distance reflects the similarity of the samples. We used PERMANOVA test to analyze the significance, p value and the F value are at the bottom right of the graph. The red represents the NC group and the blue represents the NE group.

**Figure 9:**
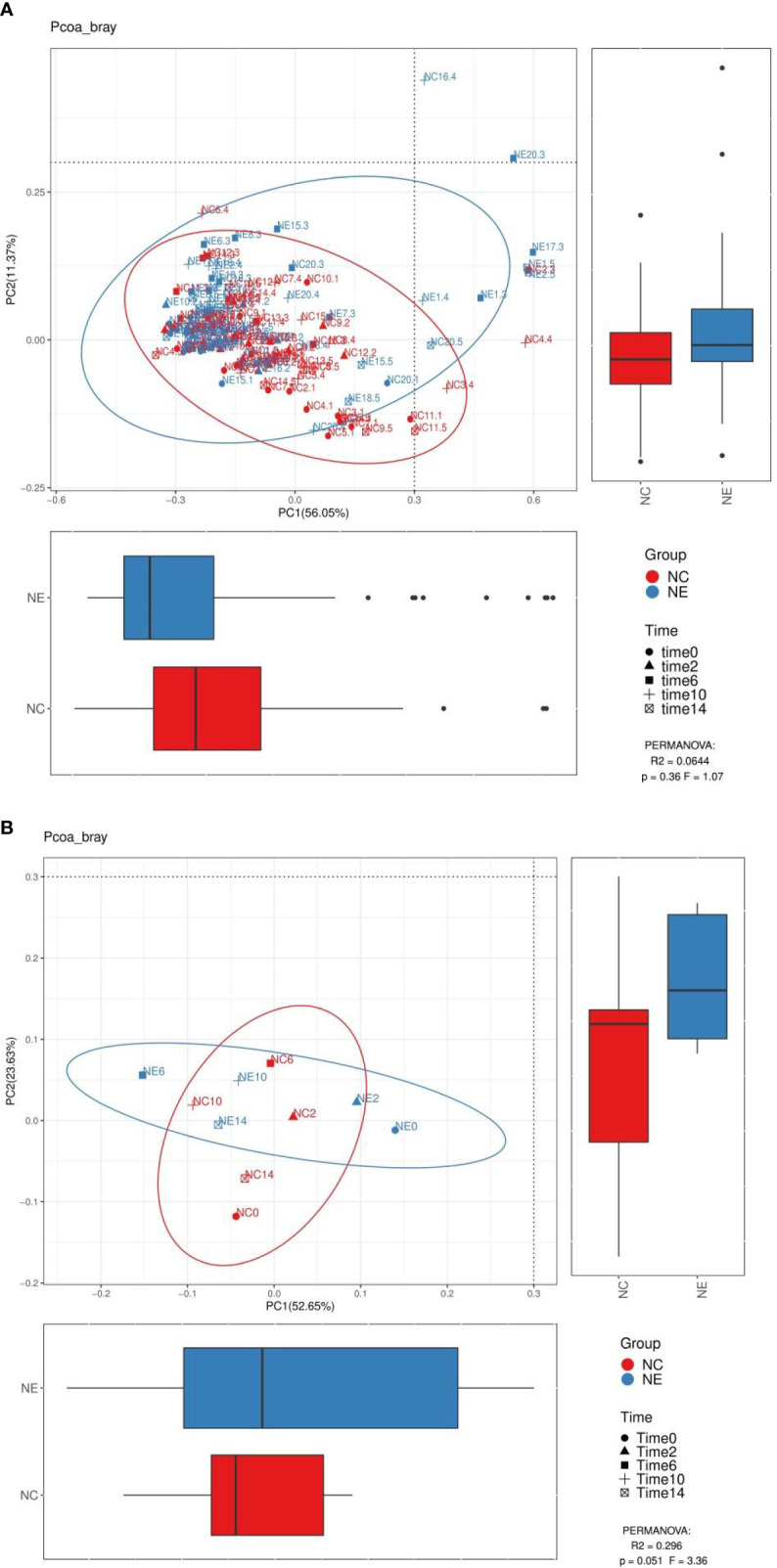
Analysis diagram of principal coordinate analysis (PCoA) at the genus level. We performed PCoA analysis based on Bray-Curtis distance. Each point in **(A)** represents a sample, and each point in **(B)** represents the average of 15 samples at a point in time. The points of the same color come from the same group, and the distance reflects the similarity of the samples. We used PERMANOVA test to analyze the significance, p value and the F value are at the bottom right of the graph. The red represents the NC group and the blue represents the NE group.

We performed PCoA analysis based on Bray-Curtis distance. As shown in **Figures 8** and **9**, there were no differences in the beta diversity of the gut microbiota between the NC and NE groups at both the phylum and genera levels, at the phylum level (**Figures 8B**), PERMANOVA: R^2^ = 0.3885, p = 0.06, F = 5.08, at the genera level (**Figures 9B**), PERMANOVA: R^2^ = 0.296, p = 0.051, F = 3.36.

### Core Bacteria and Metabolic Pathways

**Figure 10:**
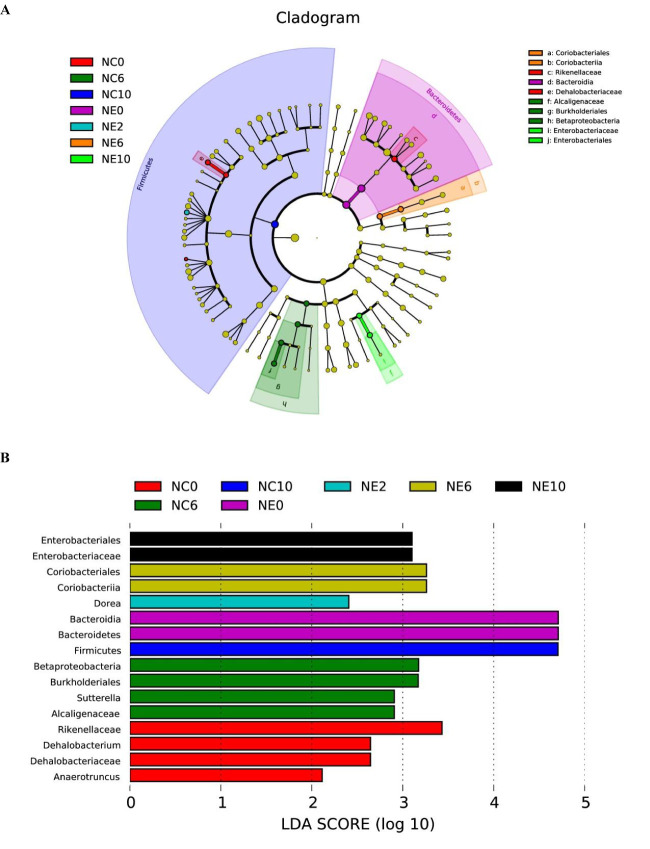
Linear discriminant analysis effect size. LEfSe was used to find the biomarker of inter-group difference. **(A)** shows the distribution of LDA values of different species. The colors represent groups. The length of the bar chart represents the contribution of different species (i.e., LDA Score). **(B)** shows the evolutionary clade of different species. The radiating circle from inside to outside represents the classification level from phylum to genus (or species).Each small circle at a different classification level represents a classification at that level, and the diameter of the small circle is proportional to the relative abundance. Coloring principle: species with no significant difference are uniformly colored in yellow, and different species are colored with the group.

**LEfSe.** LEfSe was used to find the biomarker of intergroup differences, and to find the communities or species that had significant difference on sample classification (**Figures 10**). The results showed that 16 differential biomarkers were *Anaerotruncus*, Dehalobacteriaceae, *Dehalobacterium* and Rikenellaceae of NC0; Alcaligenaceae, Sutterella, Burkholderiales and Betaproteobacteria of NC6; Firmicutes of NC10; Bacteroidetes and Bacteroidia of NE0; *Dorea* of NE2; Coriobacteriia and Coriobacteriales of NE6; Enterobacteriaceae and Enterobacteriales of NE10.

**Figure 11:**
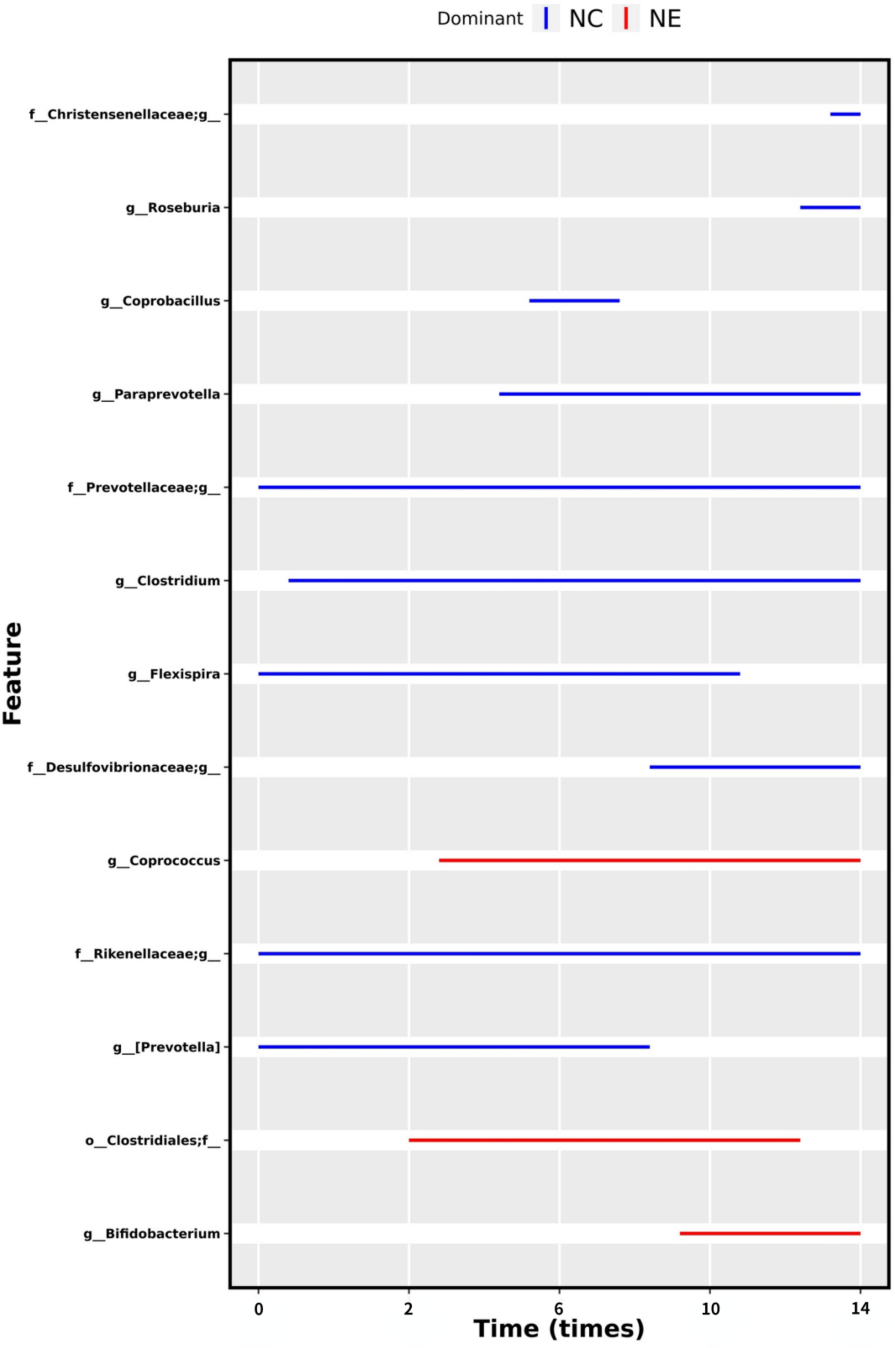
Differential bacteria under time series grouping. The time intervals of the mutually differentially abundant genera from NC and NE groups identified by MetaLonDA. Each line represents significant time interval of the corresponding bacteria. The blue lines represent the intervals where samples from the NC group have more reads, while the red lines represent the differential abundance intervals where samples from the NE group have more reads. The bacteria names are shown on the left, and the time is shown below.

We were interested in whether moderate-intensity exercise for 14 weeks resulted in the establishment of a core group of intestinal bacteria. For this reason, we used MetaLonDA to test the grouped time series data. Longitudinal analysis showed a significant time interval difference between the NC and NE groups. As shown in **Figure 11**, there were 13 significantly enriched genera in total, including *Bifidobacterium*, *Prevotella*, *Coprococcus*, *Flexispira*, *Clostridium*, *Paraprevotella*, *Coprobacillus*, *Roseburia*, one unnamed genus in the Clostridiales order, and one unnamed genus each in the Desulfovibrionaceae, Prevotellaceae, and Christensenellaceae families. Among them, the number of *Bifidobacteria*, *Coprococcus*, and one unnamed genus in the Clostridiales order was significantly increased in the NE group.

**Figure 12:**
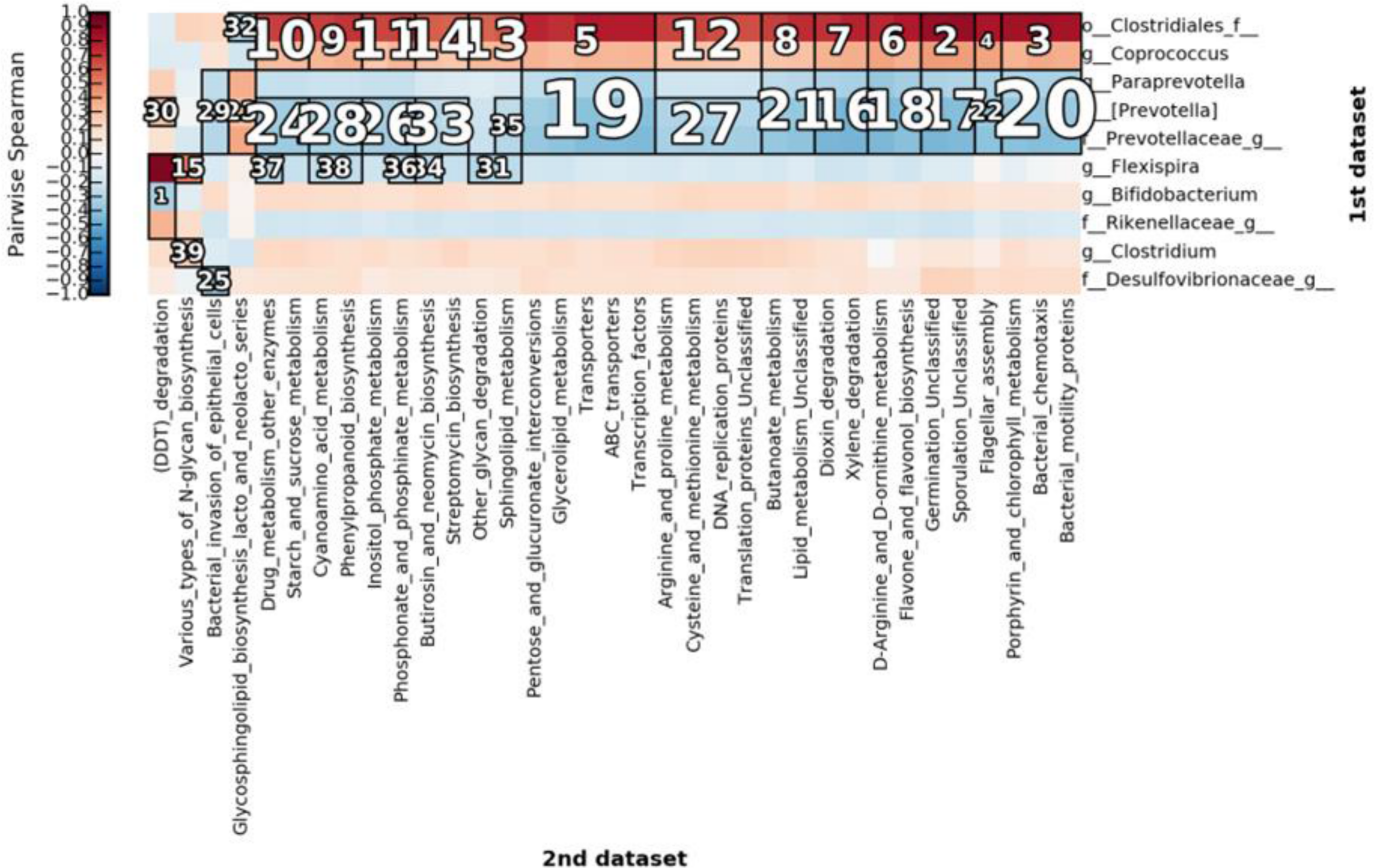
Clustering for correlation clustering analysis by HAllA. The figure highlights the correlation between clusters after clustering. The number marked in the figure is the ranking of the correlation of corresponding clusters. The smaller the number, the higher the correlation. The differential bacteria are shown on the right and metabolic pathways are shown below.

We further assessed the metabolic pathways of the core bacteria. We obtained 39 significant clusters *via* correlation cluster analysis using halla as shown in **Figure 12**. The correlation results demonstrated that most of the increased metabolic activity was related to the increase in the microbiota of the NE group and was primarily reflected in glucose metabolism, flavonoid metabolism, arginine metabolism, and proline metabolism (**Figure 12**). The metabolic pathways are shown in the **Supplementary File**.

### Correlation Analysis

The correlation analysis of exercise performance and the relative abundance of bacteria showed that exercise performance was positively correlated with the relative abundance of Phascolarctobacterium (R = 0.42, p = 0.000).

## Discussion

Previous studies have demonstrated that exercise affects the gut microbiota and microbiome in rodents **(** Matsumoto et al., 2008 **)**. Additionally, exercise has been shown to induce changes in the gut microbiome that are associated with parallel changes in host physiology **(** Queipo-Ortuño et al., 2013 **)**. While studies have indicated that exercise-induced metabolic effects have the capacity to positively reshape the gut microbiota in various animal models and humans **(** Codella et al., 2018 **)**, changes to the structure of the gut microbiota varies depending on the intensity, time, and mode of exercise **(** Mohr et al., 2020 **)**. Thus, the aim of our study was to determine if a 14-week, moderate-intensity exercise program could favorably modify the intestinal microbiota and influence the exercise performance of mice.

Our initial results found no significant difference in food intake between the two groups during the entire experimental intervention period, except during the 2nd and 14th week. The NE group consumed significantly less food than the NC group during the 2nd week, but significantly more than the NC group during the 14th week. The initial decrease in food consumption of the NE group may be related to stress, as the mice may have been adapting to formal exercise training. The increase during the 14th week may represent an increase in the caloric needs of the NE mice compared with the NC group due to formal exercise. We found no significant difference in body weight between the two groups during the experimental intervention period.

There were no significant differences in body length, Lee’s index or muscle content between the two groups, but the fat content in the NC group was significantly higher than that in the NE group. Hence, exercise can reduce fat accumulation, which is consistent with previous research results (Fu et al., 2021).

Furthermore, after 14 weeks of moderate-intensity treadmill exercise intervention, the exercise performance of mice increased significantly, and there was a difference between the NE and NC groups after 2 weeks of the exercise intervention. These results indicate that the exercise intervention scheme used in this study is effective for improving the exercise performance of mice.

In this study, we identified 11 phyla in the gut microbiota of the NE and NC groups. The three most abundant phyla in both groups were Bacteroidetes, Firmicutes, and Proteobacteria, which accounted for 97.97% of the total sequence. There were four differentially abundant phyla between the groups before intervention; these were Bacteroidetes, Firmicutes, Proteobacteria, and TM7. At week 2, the relative abundance of Actinobacteria in NC group was significantly higher than that in NE group, and at week 14, that in NC group was significantly lower than that in NE group. The effect of exercise intervention on phylum level was small, indicating that the gut microbiota was stable.

The common dominant genera of each group were *Lactobacillus*, *Allobaculum*, *Oscillospira*, *Ruminococcus*, and *Prevotella*. Before intervention, there were five differentially abundant bacterial genera, including *Lactobacillus*, *Dehalobacterium*, *Lac_[Ruminococcus]*, *Rum_Ruminococcus*, and *Desulfovibrio*. At week 2, the relative abundance of *Dorea* in NC group was significantly lower than that in NE group. At week 6, the relative abundances of *Dehalobacterium* and *Oscillospira* in NC group was significantly higher than that in NE group. At week 10, the relative abundance of *Lactobacillus* in NC group was significantly lower than that in NE group, and the relative abundances of *Oscillospira* and *Desulfovibrio* in NC group were significantly higher than those in NE group. At week 14, the relative abundances of *Bacteroides*, *Parabacteroides*, and *Odoribacter* in NC group were significantly higher than those in NE group. The results showed that the microbiota changed at the phylum level and at the genus level during different intervention periods. Similarly, other studies have found changes in the microbiome composition of mice after exercise interventions. Exercise significantly changed the overall structure and composition of microbiota **(** Matsumoto et al., 2008 **)**, the content of bacteria, such as Lactobacillus, Bifidobacterium **(** Queipo-Ortuño et al., 2013 **)**, and Proteus **(** Tan et al., 2017 **)**. The amount of exercise (total distance) was inversely proportional to the ratio of Bacteroidetes to Firmicutes, and exercise greatly increased the proportion of butyric acid-producing bacteria in the intestines of mice (Evans et al., 2014). The species diversity of the gut microbiota plays an important role in maintaining the stability and function of the intestinal ecosystem **(** Gong et al., 2016 **)**. We found that the Shannon index of NC group was significantly higher than that of NE group at weeks 6 and 10, and Chao1 of NC group was significantly higher than that of NE group at week 14. There were no significant differences in the Simpson index between the groups during the intervention period. This contrasts with previous studies that have reported that exercise may have a beneficial effect on gut microbiota diversity. One study showed that professional football players have increased gut microbiota diversity compared with ordinary people **(** Clarke et al., 2014 **)**. Another study demonstrated that after an 8-week treadmill intervention, the diversity of gut microbiota in the exercise group was significantly higher than in the normal group **(** Tan et al., 2017 **)**. Another study found that there was no significant difference in the gut microbiota diversity between sedentary wild-type C57BL/6J mice or mice that received 8 weeks of moderate-intensity treadmill intervention **(** Lamoureux et al., 2017 **)**. Hence, no consensus has been reached on the effect of exercise on the diversity of the gut microbiota in mice. This discrepancy may result from differences in mouse strains, diet, exercise intensity, exercise time, and different exercise patterns, which need to be further studied.

There was no significant difference in beta diversity between the two groups of mice in this study.

Next, we looked for markers of differences between the groups. LEfSe was used to find the biomarker of intergroup differences. The markers of NE group were *Dorea* at week 2, Coriobacteriia and Coriobacteriales at week 6, and Enterobacteriaceae and Enterobacteriales at week 10. *Dorea* is an aerogenic bacterium that uses carbohydrates to produce gas and has been linked to irritable bowel syndrome; it may be closely related to athletes often suffering from irritable bowel syndrome. It has been suggested that mice could not adapt to the exercise training in the early stage of exercise (Heiman et al., 2008).

Since we were interested in understanding if exercise altered the core members of the gut microbiota, we used MetaLonDA to perform longitudinal analysis of the data. We found a significant time interval difference between the NC and NE groups. A total of 13 genera were significantly enriched in both groups, among which the abundance of *Bifidobacteria*, Clostridia, and *Coprococcus* increased significantly in the NE group. Bifidobacterium plays an important role in maintaining microecological balance *in vivo*, can synthesize a variety of necessary vitamins, and antagonize a variety of intestinal pathogenic microorganisms. It can also control endotoxemia and improve resistance to radiation of the human body. Clostridium produces vitamins and inhibits the growth of harmful bacteria, while fecal cocci ferment carbohydrates, enhanceintestinal health and gastrointestinal function, and reduce the level of inflammation **(** Singh et al., 2017 **)**. Bifidobacterium and Clostridium decompose indigestible polysaccharides in food, thus producing short-chain fatty acids (SCFAs) such as acetic acid, butyric acid, propionic acid, and lactic acid **(** Zhao et al., 2018 **)**. Our research showed that exercise performance positively correlated with the relative abundance of Phascolarctobacterium. Phascolarctobacterium can produce SCFAs. SCFAs help certain species of microorganisms establish a dominant position in the microbiota and regulate glucose and lipid metabolism as signaling molecules. Additionally, SCFAs are primarily absorbed by intestinal epithelial cells and used for energy production. SCFAs also improve the intestinal pH and inhibit the growth of harmful bacteria such as indole-producing bacteria and hydrogen sulfide-producing bacteria within the intestinal tract **(** Singh et al., 2017 **)**.

Since exercise influenced the core members of the gut microbiota, we were interested in whether the metabolic activity of the gut microbiota was altered consequently. We found *via* correlation cluster analysis that most of the enhanced metabolic activities, mainly glucose, flavonoid, arginine, and proline metabolism, were related to the increase in the three genera in the NE group. Since exercise is supported by energy provided by the metabolism of the host, it was not surprising that these metabolic pathways were identified as being intimately associated with exercise.

Glucose is one of the main energy sources used during the process of exercise, and research has shown that glucose uptake increases in muscles during exercise due to blood flow and glucose extraction by skeletal muscle **(** McConell et al., 2012 **)**. Flavonoids have been shown to improve the level of oxidative stress in exercise-fatigued rats, inhibit body peroxidation during excessive exercise training, repair damaged cells, and promote metabolism. Increases in flavonoid metabolism are closely related to improvements in the antioxidant capacity of the body (Zhu, 2020). Arginine and proline metabolism are intimately connected to exercise fatigue (Min, 2018). Arginine is an essential amino acid that is important for protein synthesis and is also a precursor for creatine, polyamine, and nitric oxide. Arginine plays an important role in nutrient metabolism and regulation (Xu et al., 2015), and L-arginine supplementation has been shown to promote energy metabolism during exercise, affect amino acid metabolism, promote the endogenous synthesis of arginine, and support the metabolism of arginine to proline (Zhang et al., 2019). Additionally, the endurance of mice was shown to be improved by supplementation with pre-exercise carbohydrates, alanine, proline, and continuous intake of green tea catechins (Minegishi et al., 2018). Thus, our study, which is consistent with previously publishes studies, indicated that exercise and metabolism are interconnected, as enhanced metabolic activity improves exercise performance.

Thus, we speculate that the interaction between exercise and the gut microbiota affects the metabolism of the host. This conclusion is consistent with published studies. For example, one study found that an increased abundance of Prevotella was significantly correlated with not only the weekly amount of time spent exercising, but also with the number of carbohydrate and amino acid metabolism pathways **(** Petersen et al., 2017 **)**. Additionally, another study showed that professional international rugby union players exhibited relative increases in metabolic pathways, including carbohydrate and amino acid metabolism, and fecal metabolites, such as SCFAs, acetate, propionate, and butyrate. This was associated with enhanced fitness and overall health when compared with the control group **(** Barton et al., 2018 **)**. Thus, these studies and our results indicate that exercise can change the abundance of certain bacteria in the intestinal tract and the associated metabolic pathways, thereby altering the function of the body.

Our results demonstrated that moderate-intensity exercise increased the exercise performance of mice and altered the core gut bacteria, resulting in changes to metabolic pathways. However, the association between the gut microbiota and exercise performance is not clear. Evidence regarding the effect of the gut microbiota on exercise performance and physical fatigue is limited. The antioxidant enzyme system prevents oxidative damage caused by strenuous exercise and is related to the physical condition of athletes. It has been suggested that the gut microbiota may be an important environmental factor in the antioxidant defence of the host **(** Hsu et al., 2015 **)**. Therefore, the status of the gut microbiota may influence the endogenous antioxidant enzyme system of the host, while both the gut microbiota and endogenous antioxidant enzyme system of the host appear to be crucial to the performance of athletes. In addition, another study found that Veillonella proliferate in the gut of top marathoners after a race, breaking down lactic acid into propionic acid and boosting metabolism. When the bacteria were fed to mice, the exercise endurance significantly improved (Scheiman et al., 2019). Hence, while evidence supports an intimate relationship between the gut microbiota and exercise ability, the underlying mechanism of the relationship is not completely clear.

In conclusion, our results demonstrated that long-term, moderate-intensity exercise improved exercise performance and lowered the fat content in mice. The Shannon index of theNC group was higher than that of the NE group at weeks 6 and 10, and the Chao1 index was higher than that of the exercise group at week 14. Exercise performance positively correlated with the relative abundance of Phascolarctobacterium. Additionally, grouped time series data analysis showed that Bifidobacterium, Clostridium, and fecal cocci were significantly increased in the NE group, which correlated with enhanced glucose, flavonoid, arginine, and proline metabolism. Thus, moderate-intensity exercise can influence both the gut microbiota and metabolism.

These results provide a scientific reference for studying the effects of exercise intervention on the exercise performance and gut microbiota of mice. Future studies should analyze the alterations in microbiota -related metabolic enzymes induced by exercise. Identification of specific changes in the metabolic profile of mice engaged in various levels of exercise can pave the road for future efforts towards targeted manipulation of the gut microbiota and microbiome, which will provide support for the development of effective intervention programs to improve sports performance.

## Data Availability Statement

The datasets presented in this study can be found in online repositories. The names of the repository/repositories and accession number(s) can be found below: https://www.ncbi.nlm.nih.gov/, PRJNA734109.

## Ethics Statement

The animal study was reviewed and approved by School of Physical Education, South China Normal University (Guangzhou, China).

## Author Contributions

WY and YL: designing experiment. WY and GuangY: writing. ZY, GuanY, MC, and PH: data analysis. HW and XX: revising. YL and BM: project administrator. All authors contributed to the article and approved the submitted version.

## Funding

Funding for this study was received from Science and Technology Research Project of Hebei Sports Bureau (grant number: 20191020). Talent Introduction Special Funds of Lingnan Normal University to Yuqian Liu. (ZL2008) and Haitao Wang (ZL2009).

## Conflict of Interest

The authors declare that the research was conducted in the absence of any commercial or financial relationships that could be construed as a potential conflict of interest.

## Publisher’s Note

All claims expressed in this article are solely those of the authors and do not necessarily represent those of their affiliated organizations, or those of the publisher, the editors and the reviewers. Any product that may be evaluated in this article, or claim that may be made by its manufacturer, is not guaranteed or endorsed by the publisher.
