# Supplementary material for: Moderate-Intensity Physical Exercise Affects the Exercise Performance and Gut Microbiota of Mice
Source: Front Cell Infect Microbiol. 2021 Sep 24;11:712381. doi: 10.3389/fcimb.2021.712381 (PMC8498591; doi:10.3389/fcimb.2021.712381)
Supplement: Supplementary file 1 [file DataSheet_1.docx]

**Table 1**

**Summary of sample sequencing.***

|  | **Tag** | **Clean_tag** | **OTU** |
| --- | --- | --- | --- |
| Sum | 20434356 | 19742005 | 618220 |
| Min | 66738 | 57504 | 1207 |
| Max | 139999 | 138463 | 6654 |
| Mean | 136229 | 131613.4 | 4121.467 |
| SD | 13477.75 | 14672.58 | 1192.46 |

* OTU = operational taxonomic unit; SD = standard deviation.

**Table 2**

**Difference in the alpha diversity index between the control (NC) and exercise (NE) groups at the same time point.**

|  | ***p*-value** | | |
| --- | --- | --- | --- |
|  | **Shannon index** | **Simpson index** | **Chao1 index** |
| NC1 vs NE1 | 0.3568 | 0.1081 | 0.8572 |
| NC2 vs NE2 | 0.5625 | 0.9230 | 0.2957 |
| NC3 vs NE3 | 0.0674 | 0.1253 | 0.5606 |
| NC4 vs NE4 | 0.0632 | 0.3743 | 0.0470 |
| NC5 vs NE5 | 0.1061 | 0.1829 | 0.0875 |

**Table 3**

**Difference in the alpha diversity index between the control (NC) and exercise (NE) groups over time.**

|  | ***p*-value** | | |
| --- | --- | --- | --- |
|  | **Shannon index** | **Simpson index** | **Chao1 index** |
| NC1 vs. NC2 | 0.1625 | 0.3816 | 0.5668 |
| NC1 vs. NC3 | 0.1484 | 0.3472 | 0.4316 |
| NC1 vs. NC4 | 0.0184 | 0.0570 | 0.1657 |
| NC1 vs. NC5 | 0.0026 | 0.0697 | 0.0024 |
| NE1 vs. NE2 | 0.4848 | 0.6233 | 0.4864 |
| NE1 vs. NE3 | 0.0006 | 0.0144 | 0.0875 |
| NE1 vs. NE4 | 0.0001 | 0.0135 | 0.0001 |
| NE1 vs. NE5 | 0.0000 | 0.0144 | 0.0000 |

**
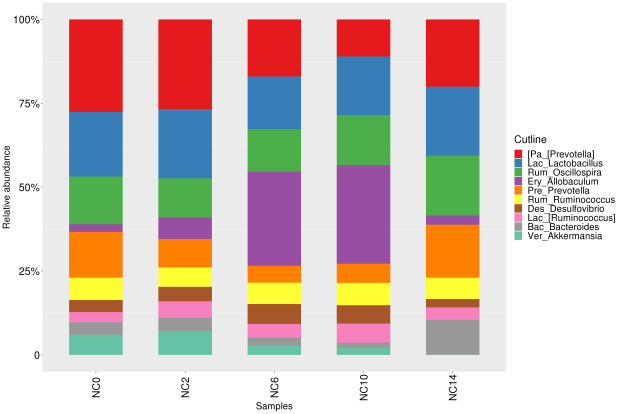
**

**Figure 1**

**
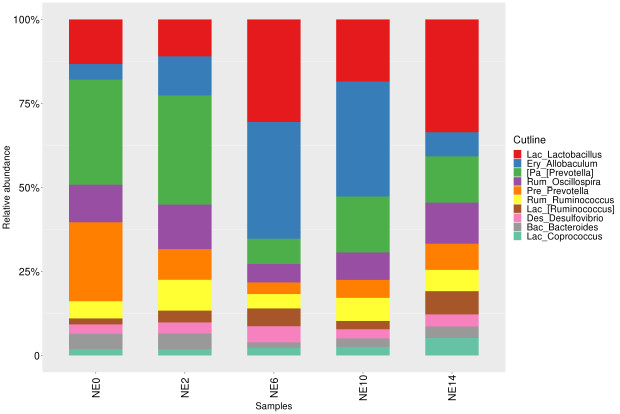
**

**Figure 2**

**Metabolic pathways:** (DDT) degradation、ABC transporters、Arginine and proline metabolism、Bacterial chemotaxis、Bacterial invasion of epithelial cells、Bacterial motility proteins、Butanoate metabolism、Butirosin and neomycin biosynthesis、Cyanoamino acid metabolism、Cysteine and methionine metabolism、D-Arginine and D-ornithine metabolism、DNA replication proteins、Dioxin degradation、Drug metabolism other enzymes、Flagellar assembly、Flavone and flavonol biosynthesis、Germination Unclassified、Glycerolipid metabolism、Glycosphingolipid biosynthesis lacto and neolacto series、Inositol phosphate metabolism、Lipid metabolism Unclassified、Other glycan degradation、Pentose and glucuronate interconversions、Phenylpropanoid biosynthesis、Phosphonate and phosphinate metabolism、Porphyrin and chlorophyll metabolism、Sphingolipid metabolism、Sporulation Unclassified、Starch and sucrose metabolism、Stilbenoid, diarylheptanoid and gingerol biosynthesis、Streptomycin biosynthesis、Transcription factors、Translation proteins Unclassified、Transporters、Various types of N-glycan biosynthesis、Xylene degradation.
